# Supplementary material for: Graptopetalum paraguayense Inhibits Liver Fibrosis by Blocking TGF-β Signaling In Vivo and In Vitro
Source: Int J Mol Sci. 2019 May 27;20(10):2592. doi: 10.3390/ijms20102592 (PMC6566198; doi:10.3390/ijms20102592)
Supplement: Supplementary file 1 [file ijms-20-02592-s001.pdf]

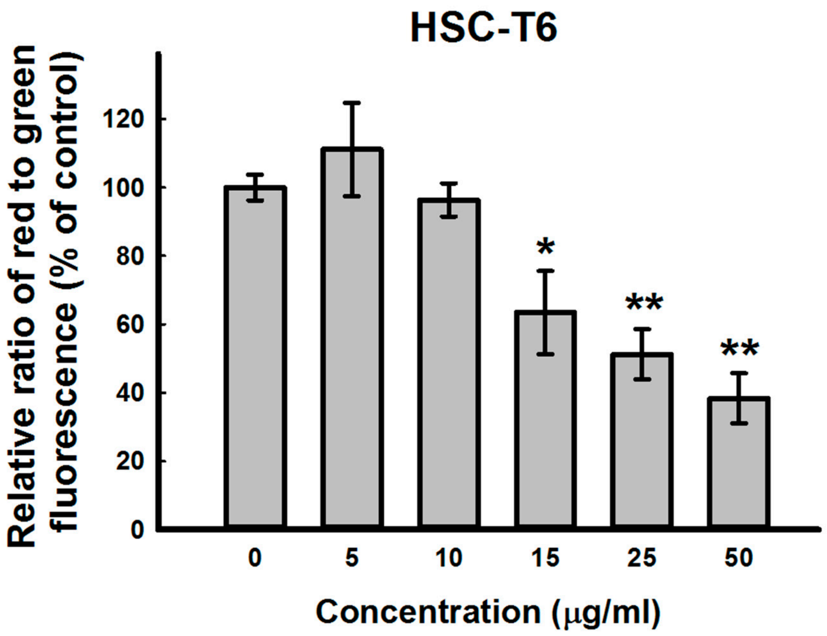

Supplementary Figure 1. Disruption of mitochondrial membrane potential in HSC-T6 cells treated with HH-F3

The mitochondrial membrane potential (ΔΨ) in HSC-T6 cells was analyzed using the JC-1 mitochondrial membrane potential assay. The ΔΨ of the cells was lower in the HSC-T6 cells treated with HH-F3 than in the control HSC-T6 cells. The treatment effect as a function of dose (0 μg/ml, 5 μg/ml, 10 μg/ml, 15 μg/ml, 25 μg/ml, and 50 μg/ml) at 48 hours is shown (n = 3).

*$P < 0.05$ compared to the control group

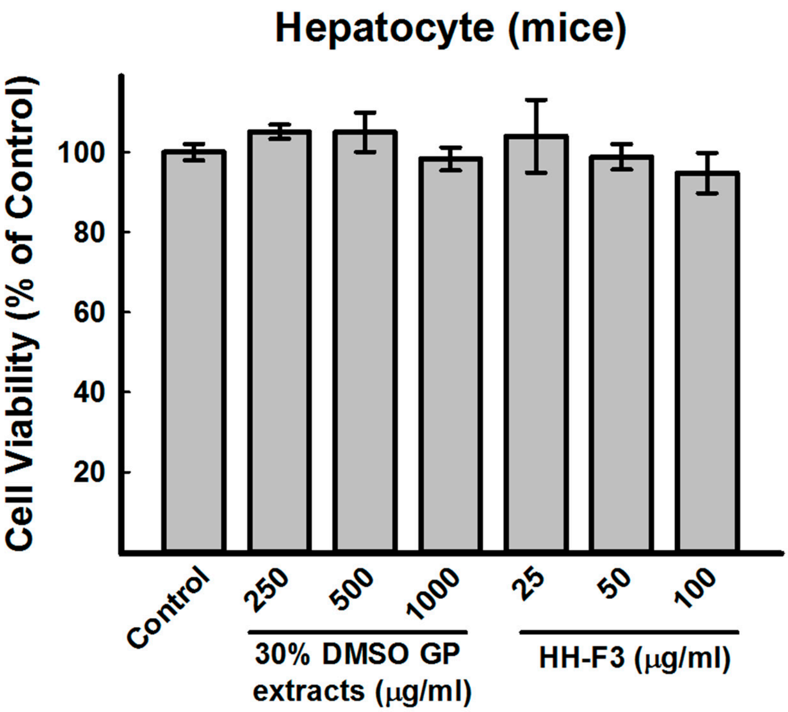

Supplementary Figure 2. The HH-F3 fraction has no effects on the growth of hepatocyte.

Mouse hepatocytes were treated with various concentrations of the 30% DMSO GP extract and HH-F3 for 24 hours and then subjected to 3-(4,5-dimethylthiazol-2-yl)-2,5-diphenyl tetrazolium bromide (MTT) assay.

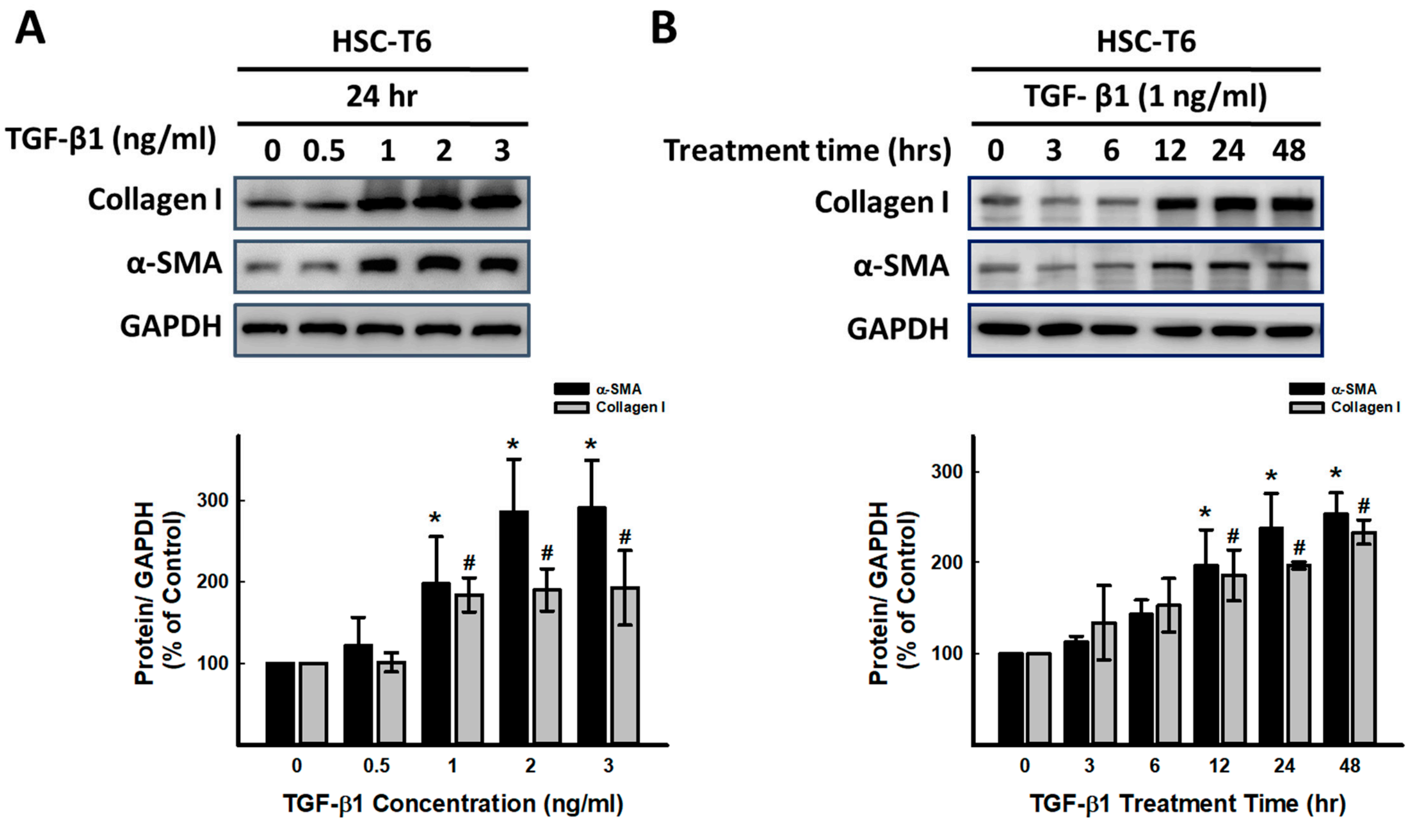

Supplementary Figure 3. TGF-β1 induces collagen I and α-SMA expression in HSC-T6 cells.

Western blot analysis and quantifications for the (A) dose (24 hours) and (B) time (TGF-β1 ng/ml)-dependent effects of TGF-β1 on α-SMA and collagen I expression. Data represent at least three independent experiments. *P < 0.05 compared to the control group (α-SMA); #P < 0.05 compared to the control group (collagen I).

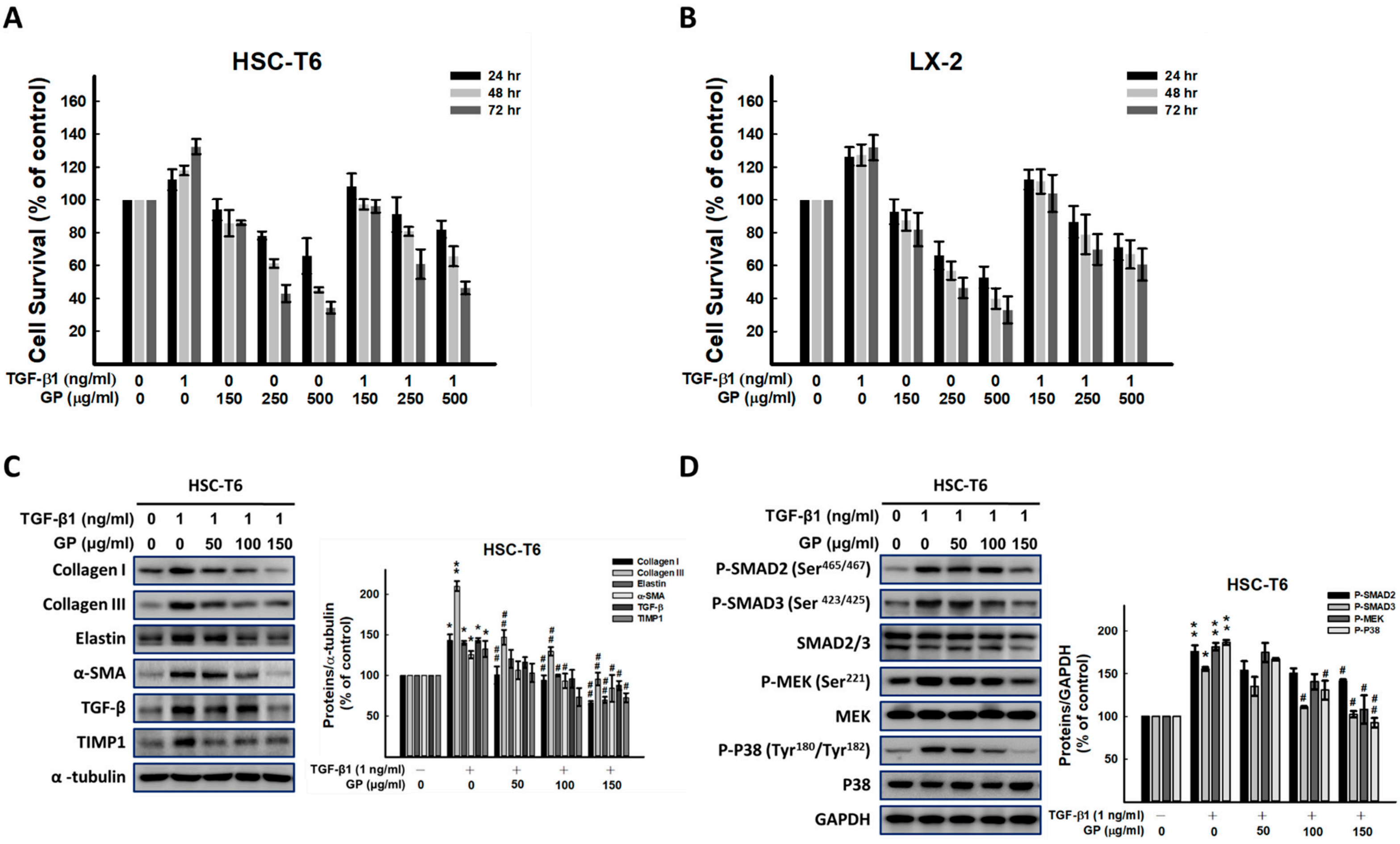

Supplementary Figure 4. The 30% DMSO GP extract inhibits TGF-β-induced cell proliferation via TGF-β pathway suppression.

(A) HSC-T6 and (B) LX-2 cell viability was assayed in the presence of either the 30% DMSO GP extract or the 30% DMSO GP extract with TGF-β (1 ng/ml) by SRB assay. (C) HSC-T6 cells were preincubated with 1 ng/ml of TGF-β for one hour and then cotreated with the 30% DMSO GP extract at different concentrations. The dose-dependent inhibitory effect on collagen type I, collagen type III, elastin, and α-SMA expression was determined by Western blot analysis. Data represent at least three independent experiments. (D) HSC-T6 cells were pretreated with 1 ng/ml of TGF-β for one hour and then cotreated with various concentrations of the 30% DMSO

GP extract for 24 hours. GP/HH-F3 block TGF-β (1 ng/ml)-induced Smad2/3, MEK, and P38 phosphorylation. Data represent at least three independent experiments. *$P < 0.05$, **$P < 0.01$ compared to the control group; #$P < 0.05$ compared to the TGF-β-induced group.

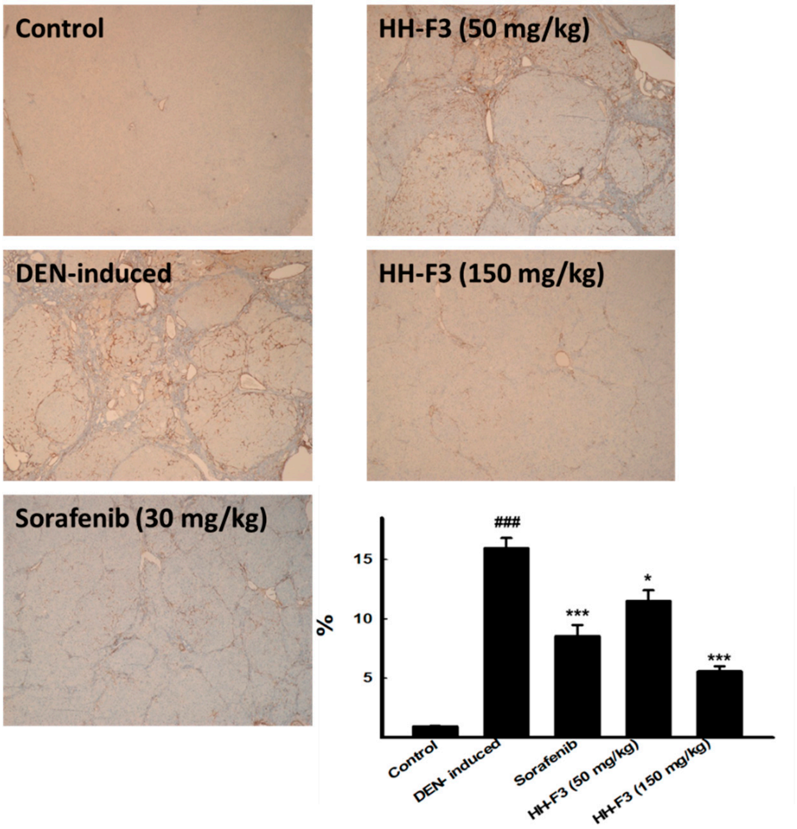

Supplementary Figure 5. Histological staining and quantitation of α-SMA in pathological sections.

In the treated rat tissue section, there are a large number of activated HSCs distributed around the portal area and in the tissues around the sinusoidal space. The rats were divided into five groups: group 1, normal control (no treatment); group 2, model group (DEN operation); group 3, sorafenib (30 mg/kg) and DEN-treated group; group 4, HH-F3 (0.05 g/kg), and DEN-treated group; group 5, HH-F3 (0.15 g/kg) and DEN-treated group. Quantitation data showed that the 150 mg/kg HH-F3 treatment group could significantly reduce α-SMA expression.

*$P < 0.05$, **$P < 0.01$, ***$P < 0.001$ compared to the DEN group; ###$P < 0.001$ compared to the control group.
